# Neural network assessment of aortic, iliac, renal, and mesenteric artery calcification in CTA: Normalized scoring framework and comparison to threshold-based method

**DOI:** 10.1177/20584601261431608

**Published:** 2026-03-05

**Authors:** Johannes Halkoaho, Oskari Niiranen, Tuomas Kaseva, Arttu Ruohola, Eero Salli, Sauli Savolainen, Harri Hakovirta, Marko Kangasniemi

**Affiliations:** 1 Department of Radiology, HUS Diagnostic Center, 3835 Helsinki University Hospital and University of Helsinki, Helsinki, Finland; 2 Department of Physics, 3835 University of Helsinki, Helsinki, Finland; 3 Department of Surgery, 8058 University of Turku, Turku, Finland; 4 Department of Vascular Surgery, Heart Center, 60652 Turku University Hospital, Turku, Finland

**Keywords:** adults, arteries, arteriosclerosis, computer applications-detection, diagnosis, CT-angiography, modalities/techniques, structures, subject matter, topics

## Abstract

**Background:**

Calcification of abdominal arteries is an important risk marker in vascular disease. Automated, objective quantification methods could improve reproducibility and reduce observer dependency in clinical practice.

**Purpose:**

To develop and evaluate a deep learning method for quantifying abdominal arterial calcification from contrast-enhanced CT angiography (CTA).

**Material and Methods:**

We retrospectively collected 223 CTA volumes, divided into 147 training and 76 test cases. Ground truth calcification segmentations were manually annotated, while vessel segmentations were generated by a previously trained neural network and manually refined. Two nnU-Net models were trained, one for artery segmentation and one for calcification segmentation. Renal, mesenteric, and common iliac arteries were shortened algorithmically. Performance of the models was evaluated using Dice score, volumetric similarity, sensitivity, precision, and Jaccard index. Calcification burden was defined as the ratio of calcified volume to artery volume. The amount and the average size of calcification clusters were investigated. The performance of the method was benchmarked against an idealized threshold-based approach and a more clinically realistic approach.

**Results:**

The neural network achieved performance comparable to the optimized threshold-based method, with slight improvements across several segmentation metrics. Dice scores and volumetric similarity demonstrated reliable vessel and calcification detection. The predicted calcification burden score showed high correlation with the ground truth calcification burden score.

**Conclusion:**

The proposed deep learning tool enables fast, reproducible, and observer-independent quantification of calcification in major abdominal vessels, offering a practical alternative to manual or threshold-based scoring methods.

## Introduction

Peripheral artery disease (PAD) affects 200 million people globally and is a major risk factor for cardiovascular mortality.^1–3^ Projections suggest a 50% increase in prevalence by 2045, with a concerning rise among younger populations.^4–7^ PAD is associated with a substantial burden of cardiovascular comorbidities and risk factors, reflecting the nature of systemic atherosclerosis.^8^ It is characterized by progressive atherosclerotic plaques within the distal aorta, iliac, and lower limb arteries. However, atherosclerosis and specific calcification of the renal and mesenteric arteries remain less well-investigated areas.

The effect of calcification of the different abdominal arteries has been widely studied and has been found to have a correlation with different comorbidities and surgical complications.^9–24^ These evaluations are commonly performed on non-contrast CT scans using the Agatston method, a well-established scoring system. The evaluation of the severity of the calcification of the arteries automatically has been done from unenhanced CT images.^25^ However, there has been an increased interest in determining calcification from contrast-enhanced CT (CTA) images.^26–28^ Since CTA is already widely used in vascular imaging, integrating calcification scoring into existing workflows could eliminate the need for additional non-contrast scans for the purpose of calcification scoring, thereby reducing patient radiation exposure.

Despite these advantages, quantifying calcification from CTA presents technical challenges. Conventional threshold-based algorithms, which are commonly used in non-enhanced scans, perform worse for CTA due to the contrast agent’s impact on Hounsfield Unit (HU) variability.^29,30^

In this study, we present a fully automated neural-network–based system for quantifying vascular calcification from CTA. Our method utilizes two neural network segmentation models; one network identifies the arteries while the second identifies the calcifications. This is supported by a post-processing algorithm that standardizes the branching lengths of the abdominal aorta and its side arteries. The neural network separates the abdominal arterial system into four clinically meaningful groups: the aorta, renal arteries, mesenteric arteries, and iliac arteries. By implementing a volume-normalized scoring framework, our system accounts for both anatomical variability and artery segmentation differences. Finally, we validate this model by comparing its performance against both idealized and clinically realistic automated thresholding methods.

The aim of this study is to establish a fast, reproducible, and observer-independent neural-network based algorithm for the calcification scoring of the abdominal aorta and its major arteries from CTA, providing a robust alternative to traditional threshold-based tools for the abdominal aorta and its major branches.

## Materials and methods

### Data

The study was authorized by the institutional review board for research (HUS Diagnostic Center decisions HUS/211/2020, 23 March 2020). The study was deemed exempt for the requirement for signed informed consent according to the Finnish research law (488/1999 and 552/2019).

A total of 223 CTA volumes, acquired between 2011 and 2013 using standard clinical protocols at Turku University Hospital, were included. The volumes were divided into a training set (*n* = 147) and a test set (*n* = 76). The test set included 12 patients with abdominal aortic aneurysms; however, patients with existing vascular stents or bypass grafts were excluded to ensure the accuracy of the automated segmentation.^31^

The distribution of scanners is summarized below.

- Siemens: Sensation 64 (test: 24, train: 51), Somatom Definition AS+ (test: 11, train: 31), and Somatom Definition Flash (train: 1).
- GE: Optima CT660 (test: 24, train: 38) and LightSpeed 16 (test: 10, train: 16).
- Toshiba: Aquilion ONE (test: 7, train: 8) and Aquilion PRIME (train: 2).

Additional relevant parameters are detailed in Table 1.

**Table 1.** Parameters for the dataset.

Parameter	Test set (*n* = 76)	Training set (*n* = 147)
Age (in years)	70 (55–90)	72 (46–93)
Gender	48 M 28 F	97 M 50 F
Tube voltage (kV)	80–120	80–120
Slice thickness (mm)	1.9 (1.0–3.0)	2.1 (0.75–3.0)
Spacing (mm)	0.8 (0.63–1.12)	0.8 (0.62–1.10)

Abbreviations: M = male; F = female; kV = kilovolt; mm = millimeter.

The age, slice thickness, and spacing are reported as a mean with the range in brackets. Tube voltage is reported as a range.

Ground truth segmentations for the arterial system were generated through a hybrid pipeline of automated prediction and expert refinement. For the training set and 21 cases in the test set, initial arterial segmentations were produced using a previous neural network.^31^ For the remaining 55 test cases, the aorta was predicted by a separate network,^32^ while the branching arteries were manually delineated. The calcification segmentations were all done manually.

All segmentations underwent manual review and correction. The segmentation was performed by a vascular surgery resident with 4 years of post-medical school graduation training with guidance and consultation from a senior radiologist with over 20 years of experience.

### Neural-network models

We employed the nnU-Net framework^33^ for 3D semantic segmentation using the default plans with the 3D full-resolution configuration. Two ensemble models were trained, each comprising five independently trained models corresponding to a five-fold cross-validation scheme. In each fold, 117–118 of the 147 total volumes were used for training, and 29–30 for validation, ensuring that each volume was used as a validation sample exactly once. All models were trained for 1000 epochs per fold using nnU-Net’s default training parameters and preprocessing pipeline. One model was trained to segment arteries and calcifications separately, assigning label 1 to arteries and label 2 to calcifications. The second model was trained to segment the aorta, iliac arteries, and abdominal branches, with the aorta assigned label 1, iliac arteries assigned label 2, the mesenteric arteries label 3, and renal arteries assigned label 4. The models were trained using Tesla v100 16/32 GB GPUs in the University of Helsinki High Performance Computing platform Turso.

### Shortening algorithm

**Figure 1.**
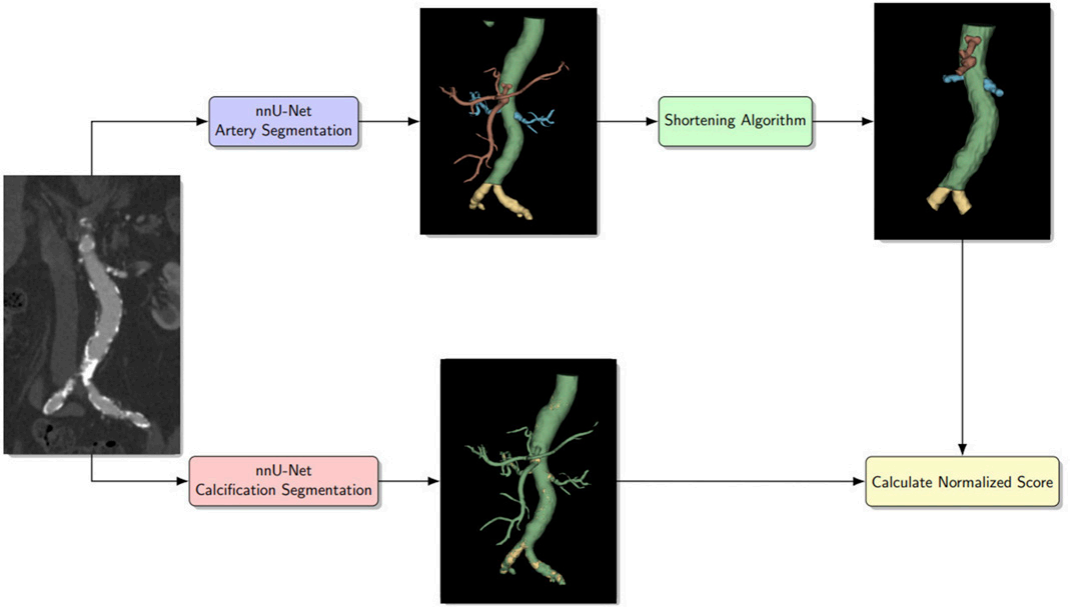
Flowchart for the method. The CTA image input is processed by two separate nnU-Net segmentation models. One segments the different arteries and the other segments the calcifications. The artery segmentation is then processed by a shortening algorithm that shortens the side arteries to a determined length (2.5 cm). Combining the shortened artery segmentation and the calcification segmentation, different metrics of interest can be determined.

To standardize the analysis across patients with varying anatomical lengths, we implemented an automated shortening algorithm as a post-processing step. After the neural network has generated a prediction for the arteries, it is then processed through an algorithm that shortens the side arteries to a determined length (2.5 cm) and truncates the aorta. It is similar to an earlier algorithm^31^ but with some improvements. The algorithm is detailed below, and the complete workflow for the method is illustrated in Figure 1.

1. Resample prediction segmentation into isotropic 1 mm spacing.
2. Run connected component analysis (CCA) on segmented side arteries.
3. For each component:
  A. Define artery stump as an intersection of the component and twice dilated aortic segmentation.
  B. Run CCA on stump, if there are components more than 2.5 cm apart from the largest components, label these components as background. Merge remaining components into a final stump segmentation.
  C. Grow each stump segmentation by dilating the stump 25 times to reach desired length. Dilated voxels not intersecting with original artery segmentation of the stump are set as background.
  D. Set label of the grown stump to match its corresponding artery segmentation.
4. Then it cuts off the aorta 1 cm above the highest side artery.

The algorithm was implemented as a Slicer module using Python 3.9.10 and Slicer 5.6.2. Additionally installed packages included scikit-image 0.24.0. A connectivity of 1 (6-connectivity in 3D) was used during CCA to define neighboring voxels.

### Threshold-based comparison methods

To compare the neural network’s performance against traditional techniques, we utilized two threshold-based approaches.

First, an idealized method was used to determine the upper bound of thresholding performance. The idealized thresholding method uses ground truth segmentation to determine the optimal global threshold on a per-case basis. The method extracts the region covered by the ground truth arteries, tests a range of global thresholds at 1% intensity intervals, and selects the one that gives the highest Dice score when comparing the thresholded result to the ground truth. Because this requires prior ground-truth knowledge, it serves as a best-case reference rather than a viable clinical tool.

Second, a clinically realistic method was implemented by calculating the mean optimal threshold (470 HU in this case) across the entire training set and applying this fixed value to the test set.

### Metrics for the neural network

Arterial segmentation performance was evaluated after the shortening algorithm was applied, using the Dice similarity coefficient, volumetric similarity, sensitivity, precision, and the Jaccard index.^34,35^

Both predicted and ground truth volumes were processed through the standardized shortening algorithm, with the ground truths manually verified and refined post-processing. Performance was evaluated across four clinically relevant regions: the aorta, iliac, renal (LRA, RRA), and mesenteric arteries (SMA, CA). Calcification segmentation performance was assessed using identical metrics but was derived from the original predictions and ground truths.

### Metrics for the calcification burden evaluation

We quantified calcification using three metrics: the total number of calcification clusters, the average cluster size, and the ratio of calcified volume to artery volume which is considered the main calcification burden score for this paper.

This patient-specific scoring framework was developed to account for the variability in arterial anatomy between patients and to mitigate the impact of fluctuations in neural network volume predictions. Because the calcification volume is anatomically constrained by the total volume of the artery, this score is naturally normalized to a range of 0 to 1.

The performance was evaluated against the ground truths for each artery group using the Pearson correlation, R^2^ correlation, the Bland-Altman plot, and a regression plot. These statistical evaluations were used across all three metrics: the normalized burden score, cluster counts, and average cluster sizes.

## Results

### Performance of artery segmentation model

**Figure 2.**
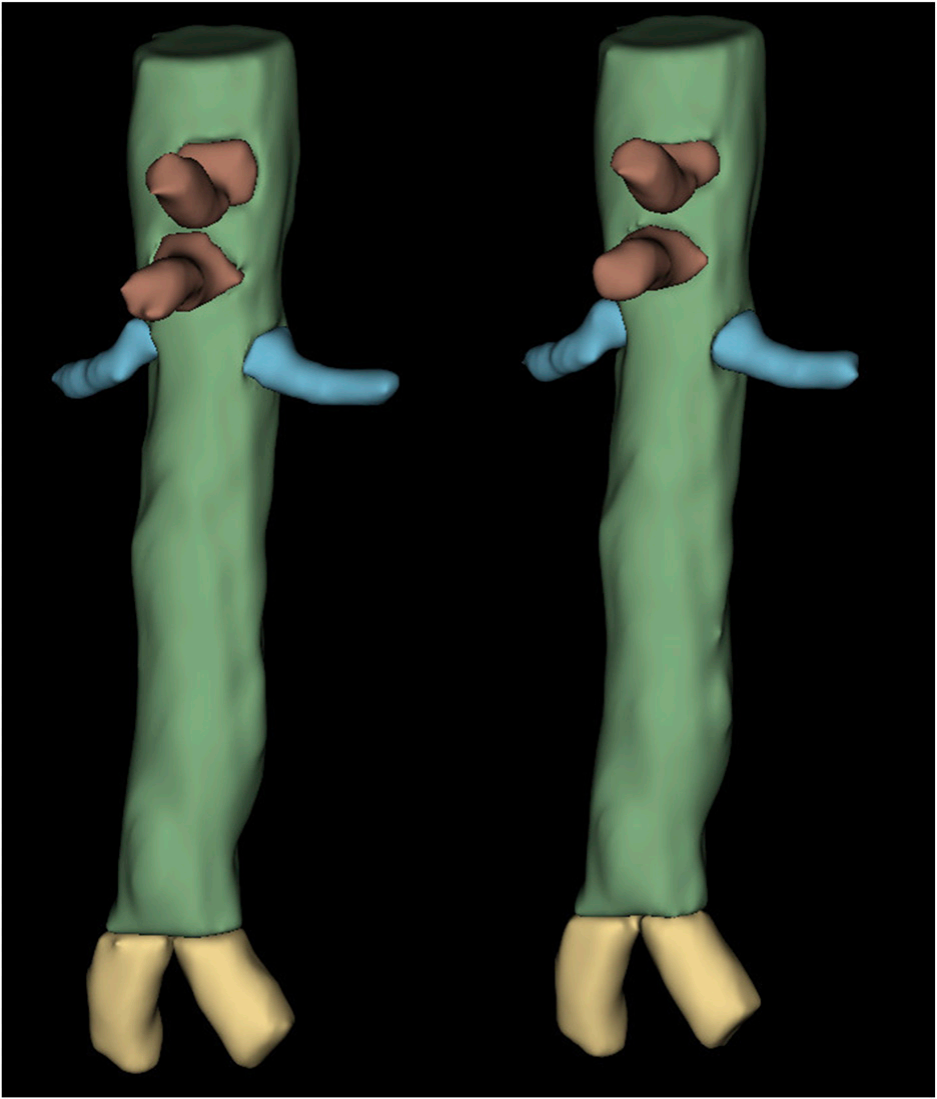
Comparison between the ground truth artery segmentation (left) and the predicted artery segmentation (right). The shortening algorithm has been used to trim the iliac, mesenteric, and renal arteries to approximately 2.5 cm.

We first evaluated the artery segmentation model after the predictions had been processed by the shortening algorithm (Table 2). The model demonstrated robust performance across all arteries, with the highest scores observed in the aorta. Performance across the other arteries (iliac, renal, and mesenteric) was consistent and comparable to one another. Overall, the scores achieved by the model provide a good foundation for the subsequent calcification burden score analysis. Figure 2 provides a visual comparison example between the ground truth and the predicted arterial segmentations.

**Table 2.** Segmentation metrics for the neural network models and the threshold-based methods.

Feature	Dice	VS	Sensitivity	Precision	Jaccard index
Abdominal aorta	0.953 ± 0.016 (0.906–0.981)	0.979 ± 0.016 (0.930–1.000)	0.959 ± 0.024 (0.867–0.991)	0.948 ± 0.033 (0.849–0.995)	0.910 ± 0.030 (0.828–0.963)
Iliac arteries	0.815 ± 0.101 (0.498–0.963)	0.933 ± 0.053 (0.711–1.000)	0.776 ± 0.112 (0.499–0.966)	0.866 ± 0.110 (0.456–0.991)	0.700 ± 0.137 (0.331–0.928)
Mesenteric arteries	0.815 ± 0.064 (0.602–0.940)	0.903 ± 0.074 (0.654–1.000)	0.883 ± 0.064 (0.667–0.984)	0.770 ± 0.117 (0.465–0.981)	0.692 ± 0.089 (0.431–0.887)
Renal arteries	0.784 ± 0.072 (0.557–0.913)	0.892 ± 0.081 (0.625–0.997)	0.841 ± 0.101 (0.411–0.989)	0.755 ± 0.119 (0.438–0.986)	0.651 ± 0.094 (0.386–0.840)
Calcification nnU-Net	0.772 ± 0.092 (0.512–0.903)	0.898 ± 0.086 (0.558–0.998)	0.771 ± 0.127 (0.360–0.958)	0.797 ± 0.123 (0.431–0.989)	0.636 ± 0.115 (0.344–0.823)
Calcification optimal thresholding	0.764 ± 0.117 (0.417–0.942)	0.887 ± 0.093 (0.655–0.997)	0.709 ± 0.162 (0.310–0.992)	0.848 ± 0.071 (0.612–0.978)	0.633 ± 0.149 (0.263–0.891)
Calcification global thresholding	0.560 ± 0.261 (0.007–0.928)	0.641 ± 0.290 (0.010–1.000)	0.676 ± 0.198 (0.213–0.985)	0.664 ± 0.357 (0.004–1.000)	0.431 ± 0.241 (0.004–0.866)

Abbreviations: VS = volumetric similarity.

The metrics are of the form mean ± standard deviation (range).

The arteries were evaluated after being processed by the shortening algorithm.

### Calcification segmentation: neural network versus thresholding

The comprehensive evaluation of the calcification can be seen in Table 2 (segmentation metrics), Table 3 (correlations), and Figures 4–7 (Bland-Altman plots and regression lines). It is important to note that while the pure segmentation metrics compare predictions directly against the calcification ground truth, the calcification burden evaluation metrics are composite measures; they are influenced by both the calcification segmentation and the accuracy of the underlying shortened arterial segmentation.

**Table 3.** Correlations between the scores (calcification burden scores, calcification cluster sizes, and counts) determined using ground truth segmentations and the scores determined using the neural network arterial predictions and the three methods for segmenting calcifications.

Feature	nnU-Net (PCC)	nnU-Net (R^2^)	Optimal thresholding (PCC)	Optimal thresholding (R^2^)	Clinical thresholding (PCC)	Clinical thresholding (R^2^)
Aorta calcification burden score	0.955	0.907	0.958	0.885	0.162	−19.8
Iliac arteries calcification burden score	0.943	0.888	0.932	0.855	0.443	−0.987
Mesenteric arteries calcification burden score	0.869	0.654	0.891	0.705	0.216	−9.13
Renal arteries calcification burden score	0.933	0.773	0.930	0.806	0.417	−5.45
All arteries combined calcification burden score	0.954	0.905	0.958	0.887	0.187	−13.3
Aorta calcification cluster count	0.781	0.445	0.607	−8.13	0.118	−53.0
Iliac arteries calcification cluster count	0.802	0.590	0.779	0.353	0.302	−14.7
Mesenteric arteries calcification cluster count	0.691	0.464	0.508	−4.05	0.107	−28.3
Renal arteries calcification cluster count	0.800	0.636	0.771	0.353	0.191	−9.09
Aorta average calcification cluster size	0.526	−7.76	0.59	−0.037	0.059	−2094
Iliac arteries average calcification cluster size	0.445	−0.16	0.48	0.123	0.165	−2.35
Mesenteric arteries average calcification cluster size	0.926	0.673	0.654	0.422	0.187	−4.02
Renal arteries average calcification cluster size	0.803	0.024	0.776	−0.118	0.466	−16.8

Abbreviations: PCC = Pearson correlation coefficient.

The scores here are composite scores utilizing both arterial and calcification segmentations.

**Figure 3.**
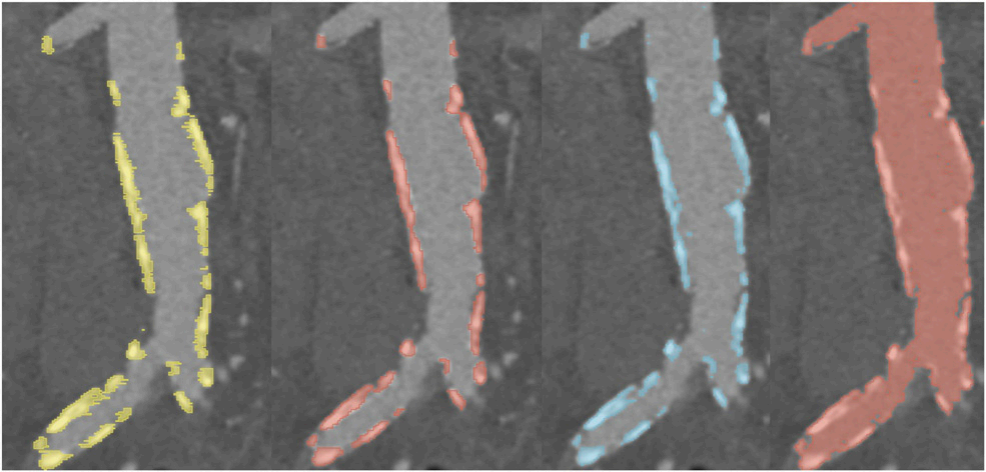
Comparison between the ground truth calcification segmentation (left) and the predicted segmentation by different methods (neural network prediction middle left, optimized threshold middle right, and the clinical threshold on the right). This illustrates the limitation of the clinical method as the HU values of the arteries were higher than 470 HU.

**Figure 4.**
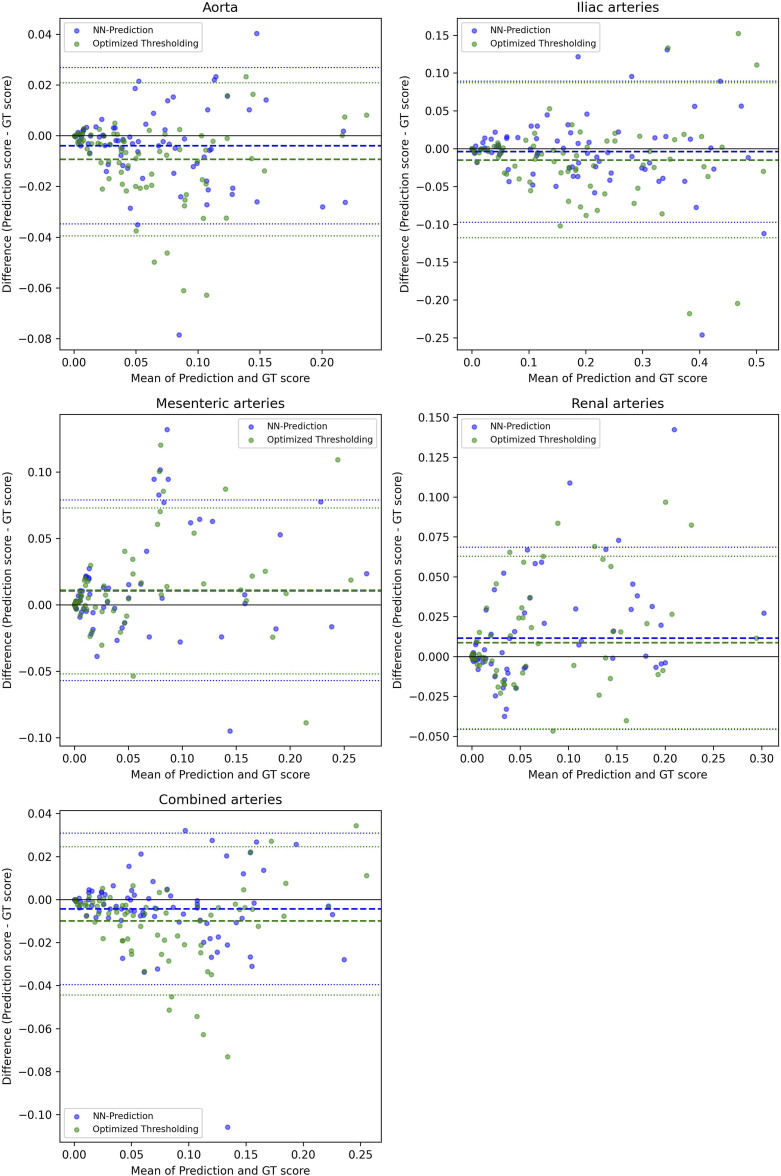
Bland-Altman plots for the datapoints. It shows the plots for the scores of different arteries; aorta, iliac arteries, mesenteric arteries, and renal arteries and then the combined score of the arteries between the ground truth scores and both the optimal thresholding method score and the neural network method score. The score is the volume of calcification divided by the volume of the artery. The dotted middle line shows the mean difference between the ground truth score and the predicted score, and the outer dotted lines show the mean difference ±1.96*SD (SD = standard deviation of the difference).

**Figure 5.**
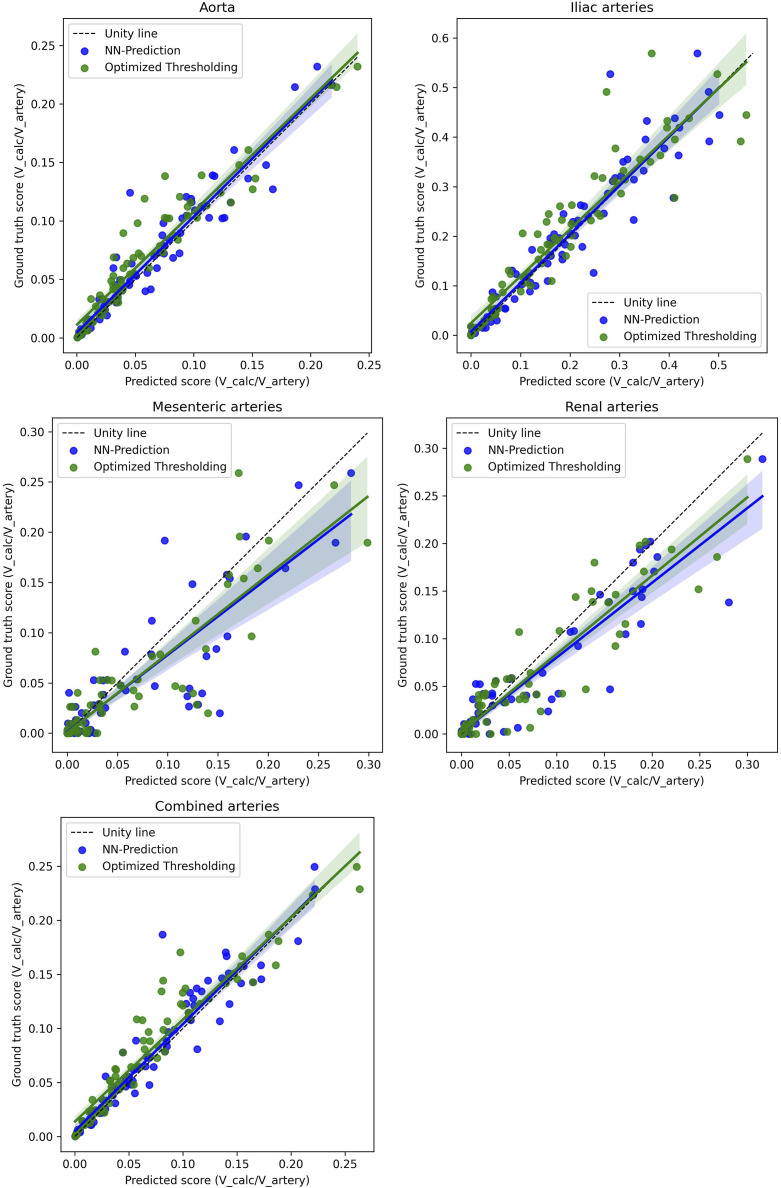
The figure shows linear regression plots for the calcification scores of different arteries; aorta, iliac arteries, mesenteric arteries, and renal arteries and then the combined score of the arteries between the ground truth and the predicted score values for both the optimal thresholding method score and the neural network method score. The score is the volume of calcification divided by the volume of the artery. The colored area represents 95% confidence intervals, and the dotted line represents the line of unity. In Seaborn, the confidence interval is estimated using a bootstrap.

**Figure 6.**
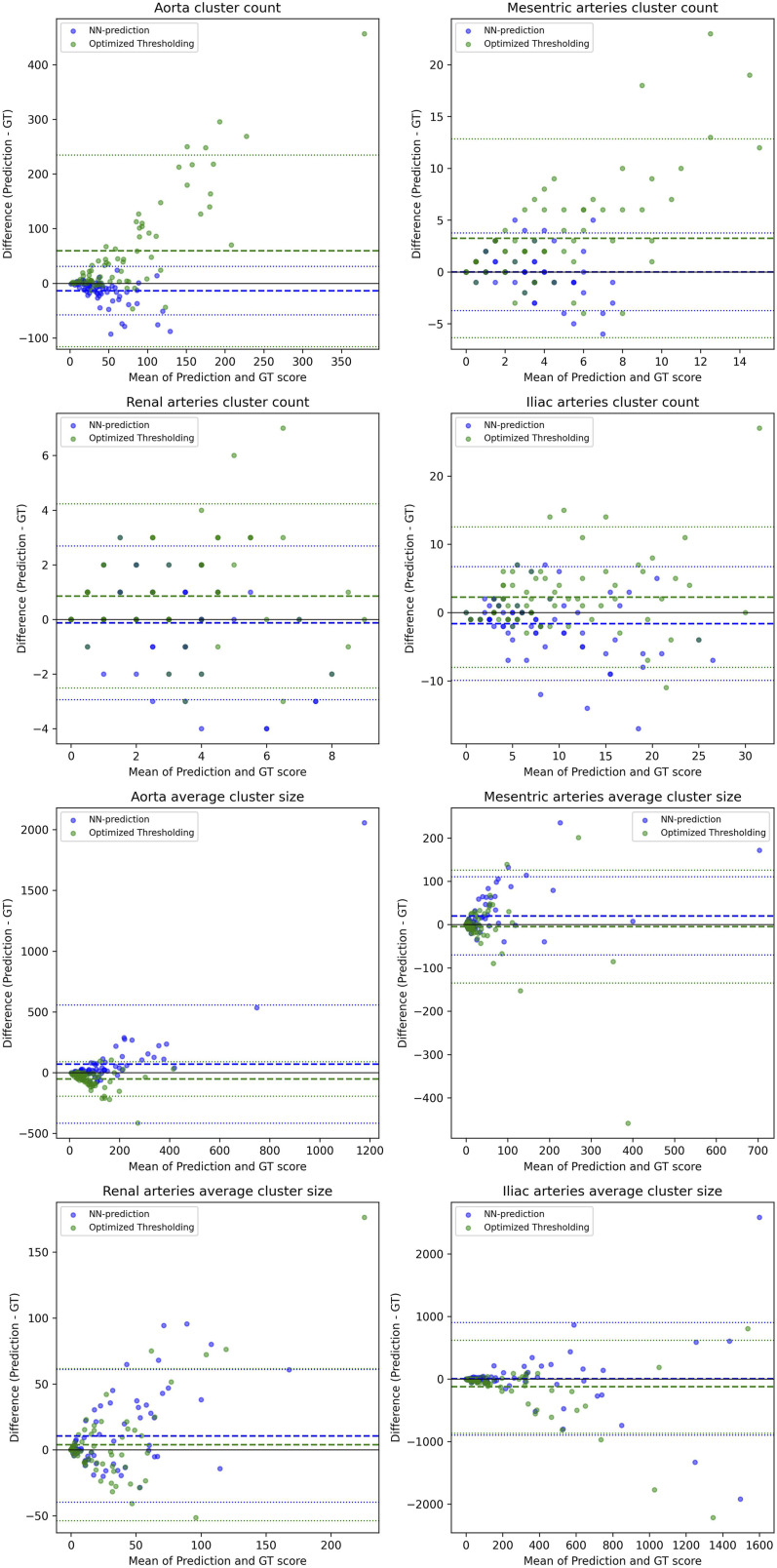
Bland-Altman plots for the calcification clusters. It shows the plots for the amount and sizes of calcification clusters of different arteries; aorta, iliac arteries, mesenteric arteries, and renal arteries between the ground truth clusters and both the optimal thresholding method clusters and the neural network method clusters. The average size of the clusters is in mm^3. The dotted middle line shows the mean difference between the ground truth score and the predicted score, and the outer dotted lines show the mean difference ±1.96*SD (SD = standard deviation of the difference).

**Figure 7.**
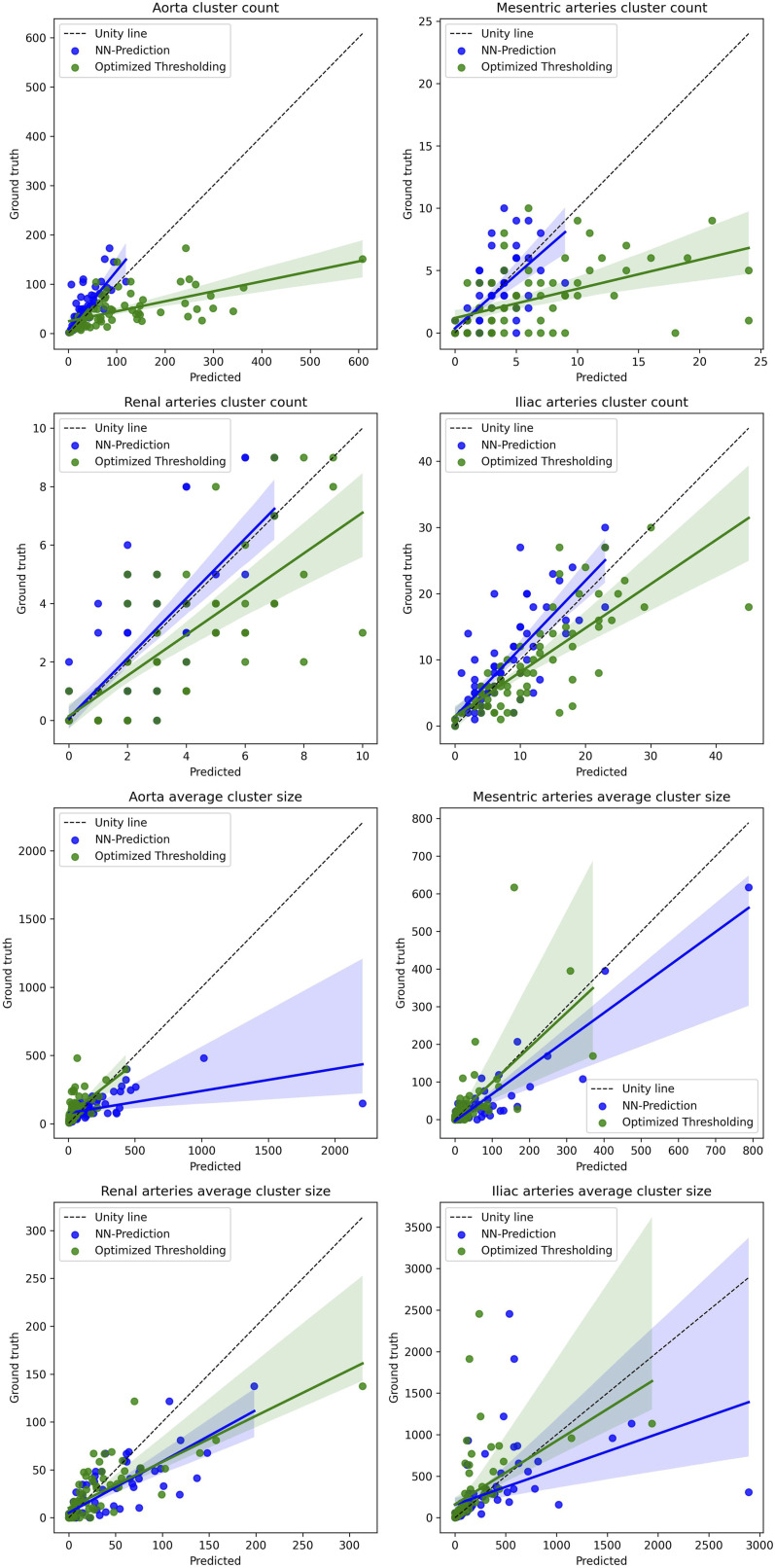
The figure shows linear regression plots for the amount and sizes of calcification clusters of different arteries; aorta, iliac arteries, mesenteric arteries, and renal arteries between the ground truth clusters and both the optimal thresholding method clusters and the neural network method clusters. The average size of the clusters is in mm^3. The colored area represents 95% confidence intervals, and the dotted line represents the line of unity. In Seaborn, the confidence interval is estimated using a bootstrap.

In terms of segmentation metrics (Table 2), the neural network method slightly outperformed the optimized thresholding method in most categories. The notable exception was precision, where the optimized thresholding method was superior, whereas the neural network demonstrated greater sensitivity. The neural network seems to offer a more balanced compromise between sensitivity and precision. In contrast, the clinically realistic thresholding method failed to reach the performance levels of either the neural network or the optimized approach (Tables 2 and 3). A visual comparison between the calcification segmentations can be seen in Figure 3.

Next is the analysis of the accuracy of the calcification burden score. Bland-Altman analysis (Figure 4) revealed that both the neural network and optimized thresholding methods tended to underestimate calcification scores in the aorta and iliac arteries, though the bias was more pronounced in the optimized thresholding method. Conversely, in the smaller mesenteric and renal arteries, the bias shifted toward overestimation. Despite these trends, most data points for both methods fell well within the confidence intervals.

Regression analysis (Figure 5) showed that the neural network’s predictions for the aorta and iliac arteries aligned closely with the unity line. Although the correlation was slightly lower in the smaller side arteries (mesenteric and renal), the combined artery volume score had high correlation as it is dominated by the larger aortic and iliac volumes.

While the optimized thresholding method generally showed slightly higher Pearson correlation coefficients (PCCs) in Table 3 than the neural network (except in the iliac arteries), a paired t-test confirmed no statistically significant difference between the two methods regarding either Dice scores or final calcification scores (*p* > .05). Overall, the high PCC for the calcification burden score across all arteries supports the feasibility of this method for the evaluation of the calcification burden.

Finally, we analyzed the morphology of the calcifications (Figures 6 and 7). The models exhibited distinct behaviors: the optimized thresholding tended to overestimate the amount of calcification clusters. Conversely, the neural network tended to underestimate the count while overestimating the average size of clusters. This suggests that the network merges adjacent small calcifications into single, larger clusters. In Table 3, we see the correlation between the prediction and the ground truths favors the neural network model in the cluster counts and generally the optimized thresholding method in cluster sizes.

The correlations for cluster counts and sizes were significantly weaker than those for the total calcification score (Table 3). This finding supports the use of the volume-normalized burden score as a more robust and reproducible metric for the evaluation of the calcification burden.

## Discussion

This study compared a neural network-based method for quantifying aortic, iliac, renal, and mesenteric artery calcification with an idealized threshold-based approach and a more clinically achievable automated thresholding method. Our results demonstrate that the neural network and idealized thresholding achieved similar outcomes, with high correlations to the ground truth, while the automated clinical thresholding performed significantly worse. This suggests that a deep-learning approach can match or even slightly improve upon idealized thresholding, indicating the feasibility of automated calcification scoring in CTA. Consequently, this method offers a potential alternative to traditional non-contrast Agatston scoring,^25^ potentially reducing patient radiation exposure by eliminating the need for additional unenhanced scans.

We utilized separate neural networks to segment calcifications and arteries to better determine which artery the calcification belongs to. In the case where the model would have predicted both it would be more difficult to make that determination, especially on the borders of the different arteries. We opted for a post-processing shortening algorithm over training on pre-shortened data mostly due to its inherent flexibility; for example, the length of side arteries can be adjusted from 2.5 cm to 3.5 cm without requiring model retraining. Furthermore, to account for inter-patient anatomical variability and variations in artery segmentation quality, we implemented a volume-normalized scoring framework that enables more consistent comparison of calcification burden across different artery types and patients. While both artery and calcification segmentations influence the final score, normalization by artery volume helps reduce the bias caused by the artery segmentation.

In addition to volumetric quantification of the calcification, we also evaluated the number and average size of calcification clusters within each artery group. This analysis provides a complementary perspective on the morphology of vascular calcification, which may have different clinical relevance compared to pure calcification volume. However, the correlations suggest that the current method is not optimal for consistently and accurately evaluating cluster sizes and counts.

The failure of the simple clinical thresholding algorithm further illustrates the need for case-specific methods. We tested more nuanced approaches, such as Otsu-thresholding and highest-HU percentiles, but neither improved Dice scores. A primary reason for the poor correlation in clinical thresholding was that in cases where the entirety of the artery had higher than average HU values, the algorithm occasionally misidentified the entire artery as calcification (Figure 3).

A key element of this study is the division of the abdominal arterial system into four anatomically and clinically relevant groups: the aorta, iliac arteries, renal arteries, and mesenteric arteries (coeliac and superior mesenteric arteries). This separation enables more detailed, region-specific analysis of calcification patterns and reflects the varying clinical implications across vascular territories. By consistently labeling and standardizing the segment lengths of these arteries, our method supports the development of more precise inter-patient comparisons and artery-specific or composite risk scores tailored to clinical context.

Despite these findings, some limitations of this study must be noted. Both the training and the test data were obtained from a single center, leaving the model’s generalizability to other patient populations unclear. Vascular stents and bypass grafts, which can interfere with calcification and artery segmentation,^31^ were excluded from the test set. However, future improvements in imaging, such as the photon-counting detectors, may eventually allow for easier differentiation between stents and calcifications.^36^ Moreover, the clinical relevance of the calcification burden score requires further validation before it can be applied in clinical practice. While the method currently provides four artery-specific scores, the next possible step would be to create a composite calcification score potentially with weighted contributions based on the clinical significance of each territory. Further research is necessary to validate the correlation and predictive value of the artery-specific scores regarding clinical outcomes. These scores hold significant potential as predictive markers for complications such as renal insufficiency, mesenteric ischemia, and postoperative bowel resection complications, warranting further investigations. The predictive value of different factors has been studied previously using machine learning methods.^37^

In conclusion, our results demonstrate that a neural network-based system offers a robust, observer-independent alternative to threshold-based techniques for calcification assessment in CTA. With further validation and clinical integration, this method may enhance cardiovascular risk assessment and support treatment planning in patients with peripheral artery disease.

## Data Availability

The datasets analyzed during the current study are not publicly available due to Finland’s regulations, but some parts of the analysis are available from the corresponding author on reasonable request*.
